# Supplementary material for: Preparation, Properties and Cell Biocompatibility of Room Temperature LCST-Hydrogels Based on Thermoresponsive PEO Stars
Source: Gels. 2021 Jul 6;7(3):84. doi: 10.3390/gels7030084 (PMC8293143; doi:10.3390/gels7030084)
Supplement: Supplementary file 1 [file gels-07-00084-s001.zip › gels-1265943-supplementary.pdf]

Supplementary Files.

Figure 1 NMR and IR of Polymer P1

Figure 2 NMR and IR of Polymer P2

Figure 3 NMR and IR of Polymer P3

Figure 4 NMR and IR of Polymer P4

Figure 5 NMR and IR of Polymer P5

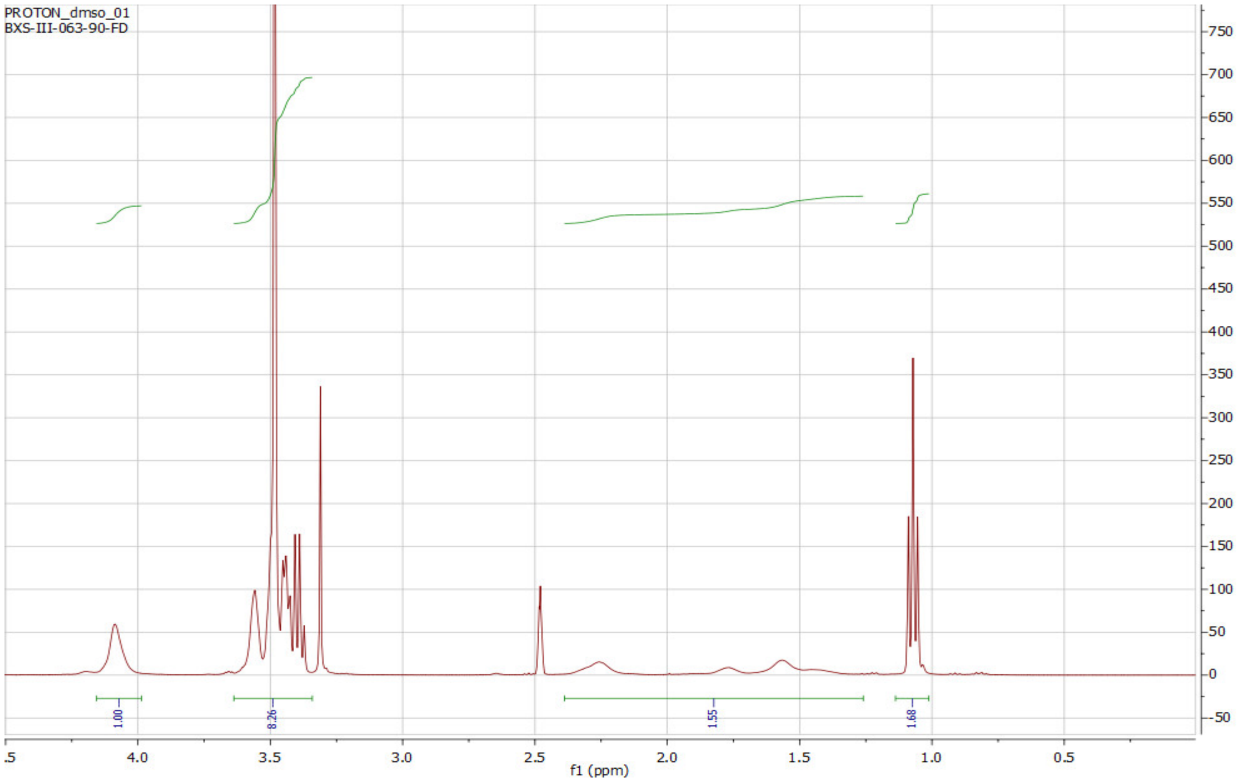

Figure S1a NMR spectra of polymer P1 in DMSO-D6

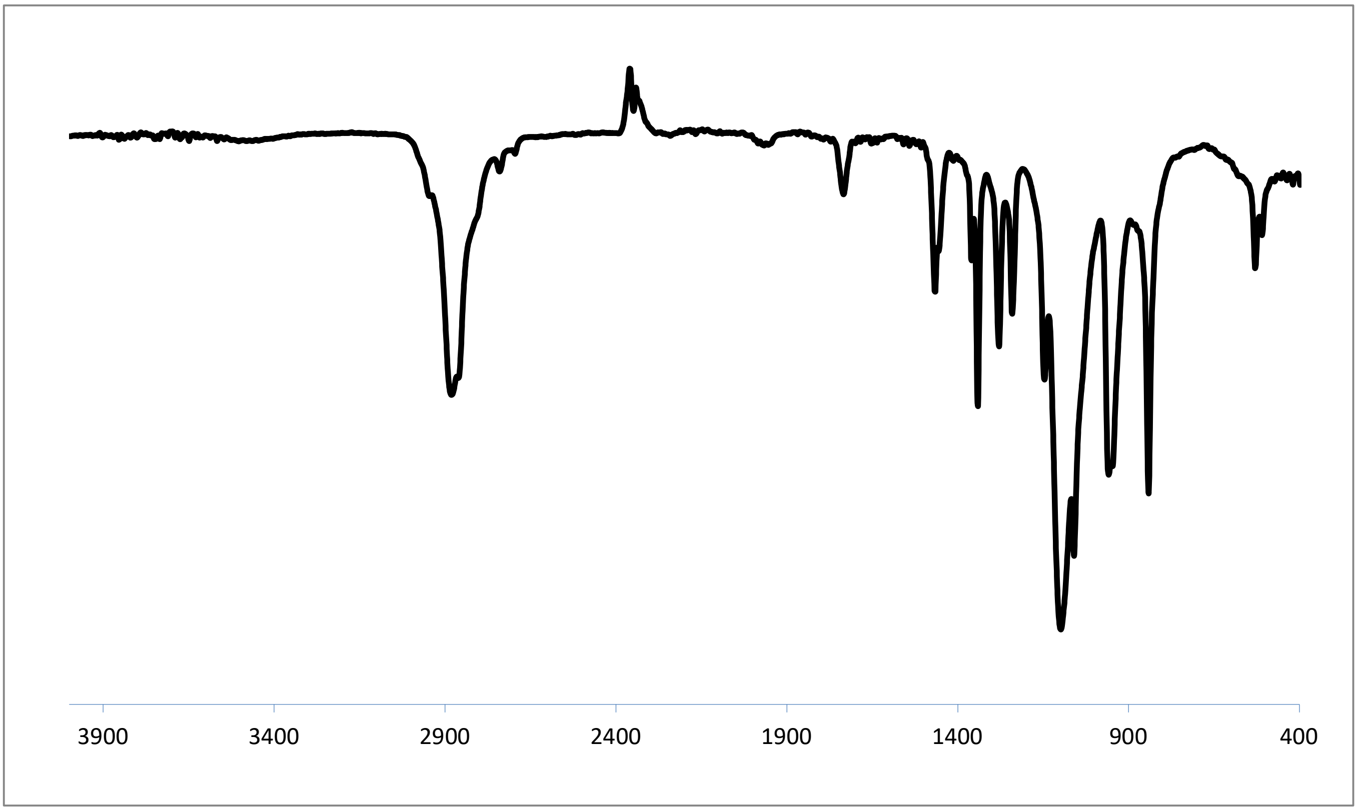

Figure S1b IR spectra of Polymer S1 (neat)

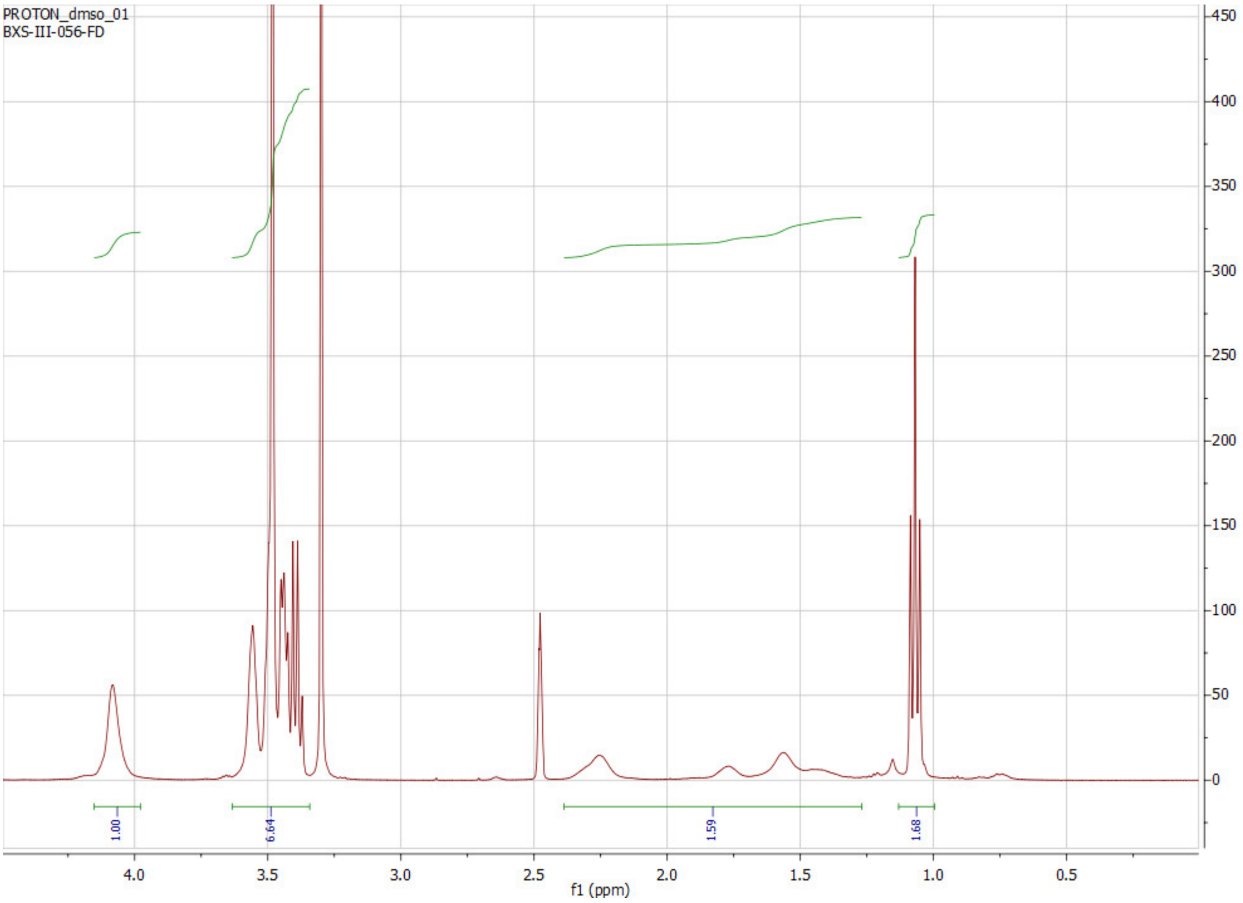

Figure S2a NMR spectrum of P2 (DMSO-D6)

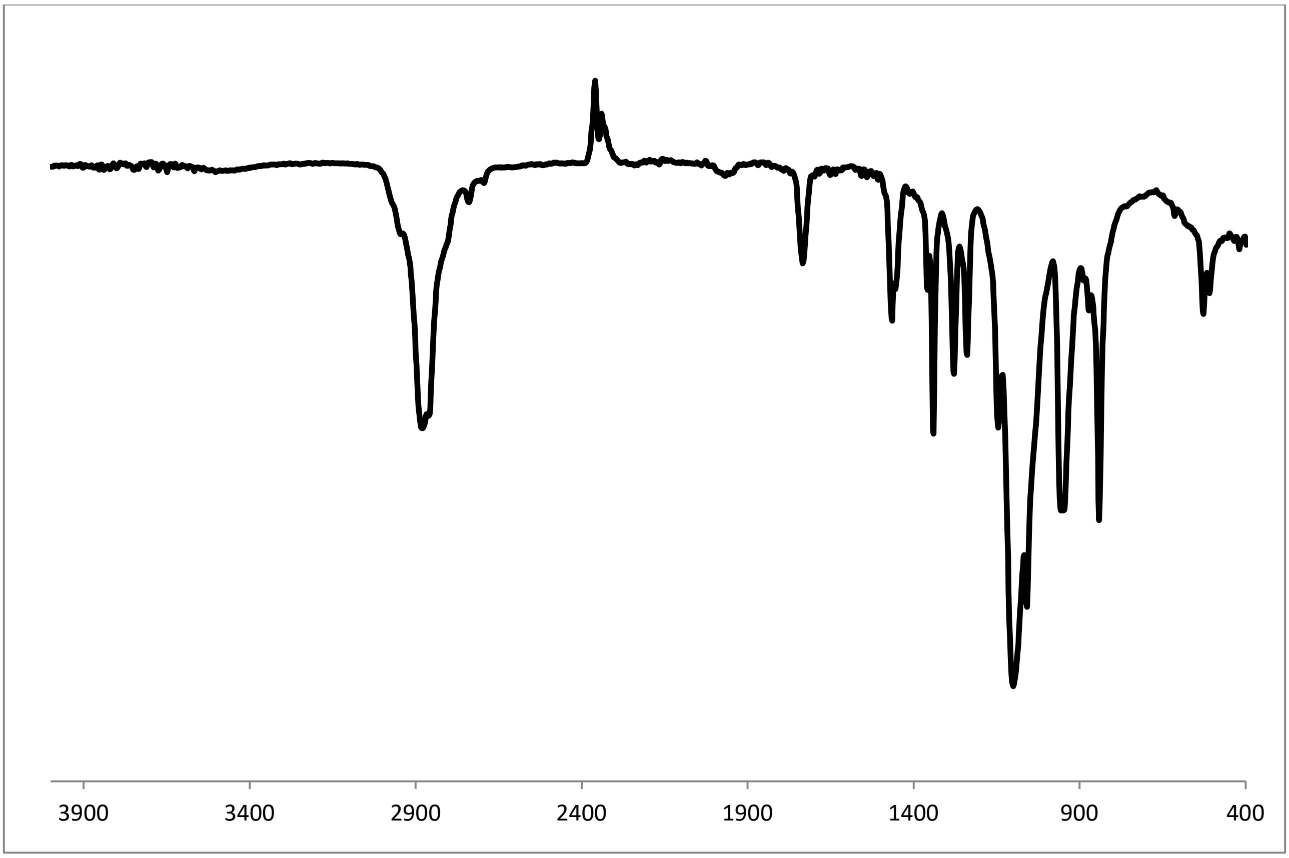

Figure S2b IR spectrum of P2 (neat)

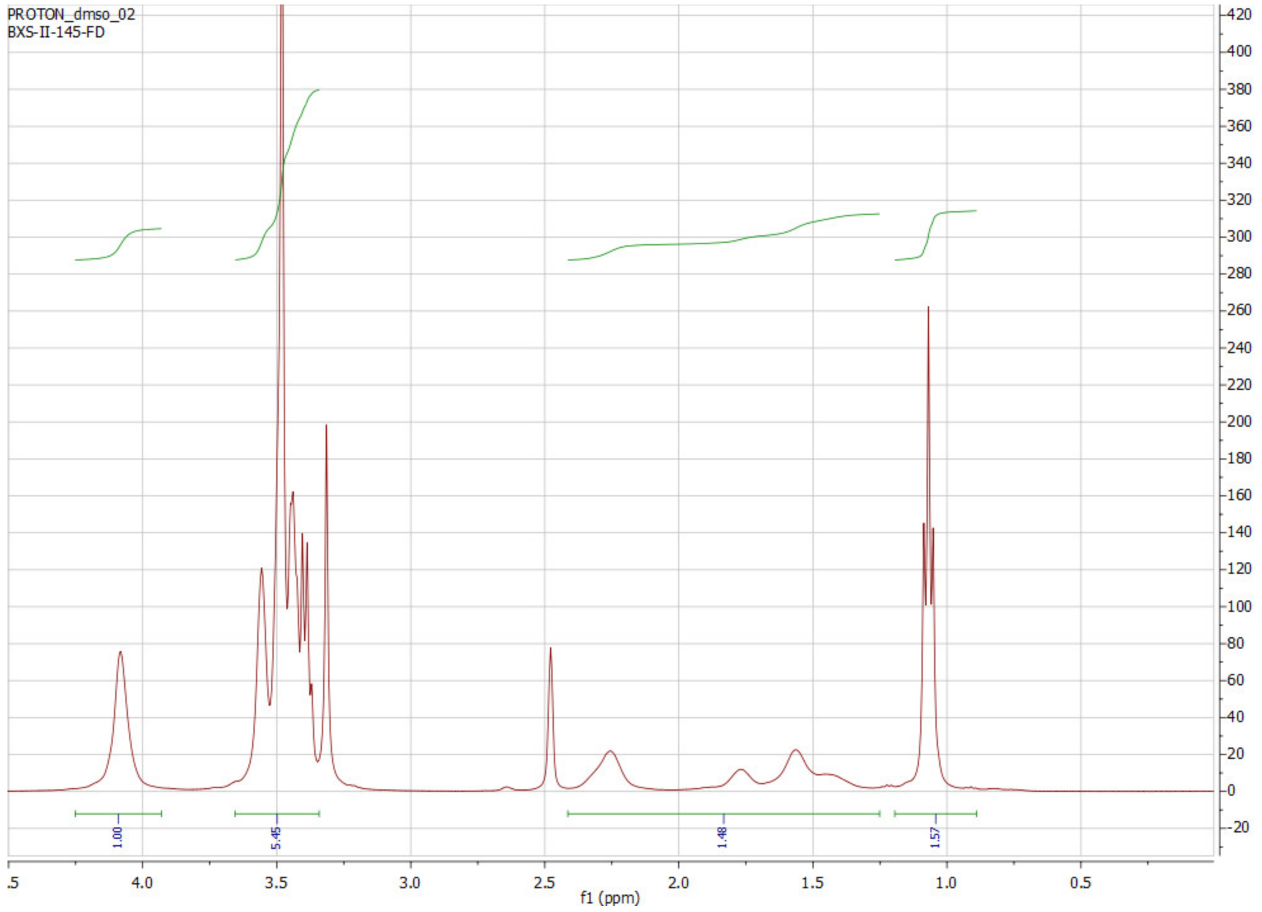

Figure S3a NMR spectrum of P3 (DMSO-D6)

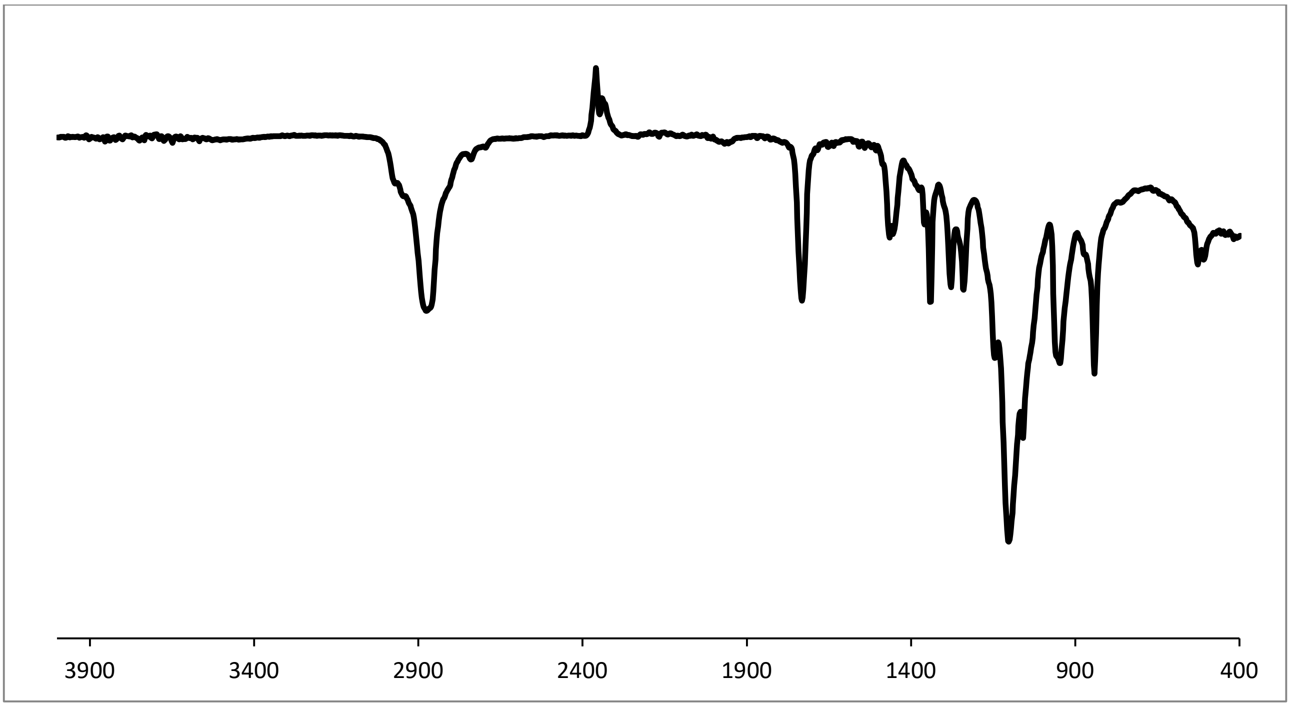

Fig 3b IR spectrum of P3 (neat)

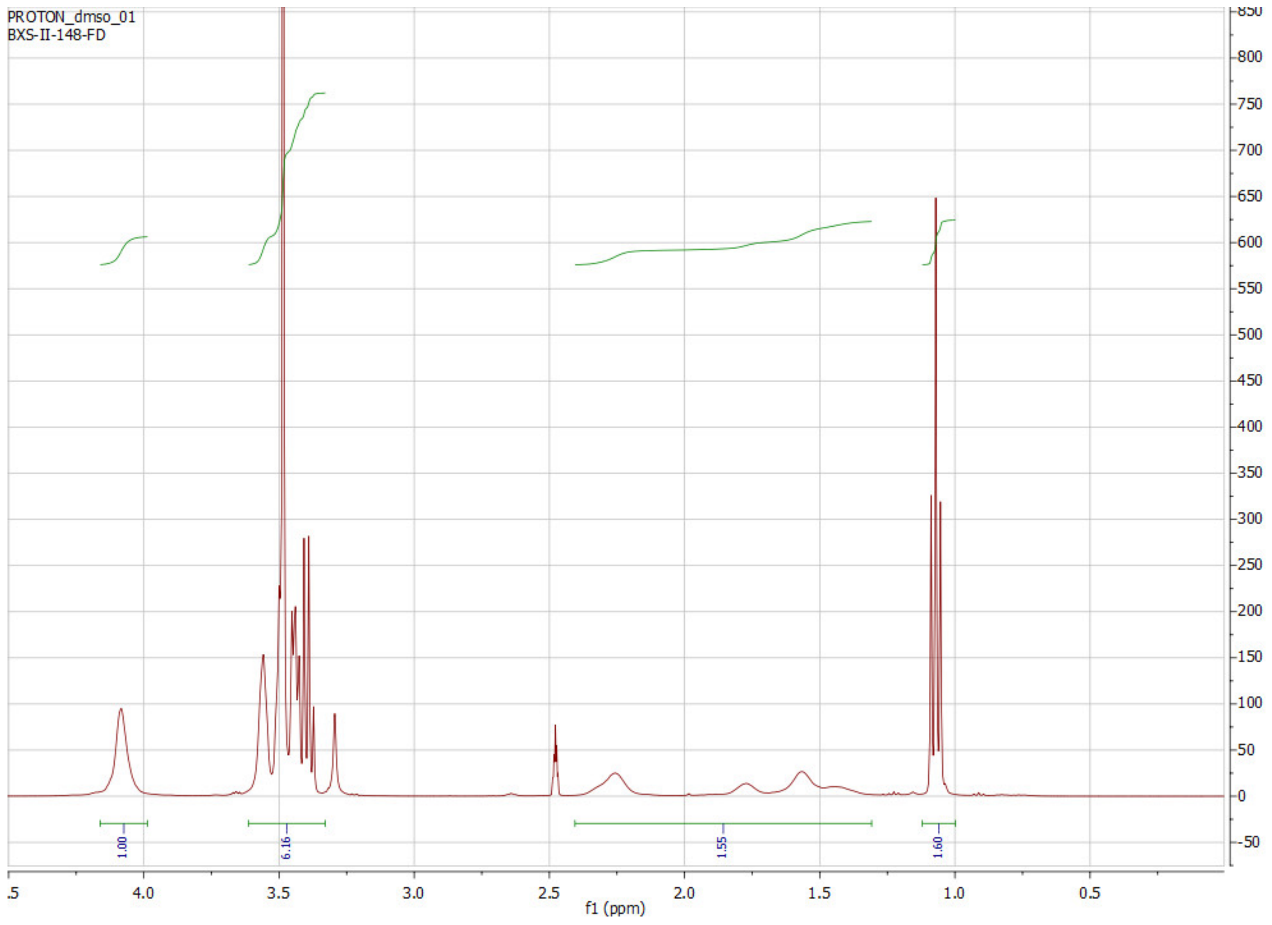

Figure 4a NMR spectrum of P4 (DMSO-D6)

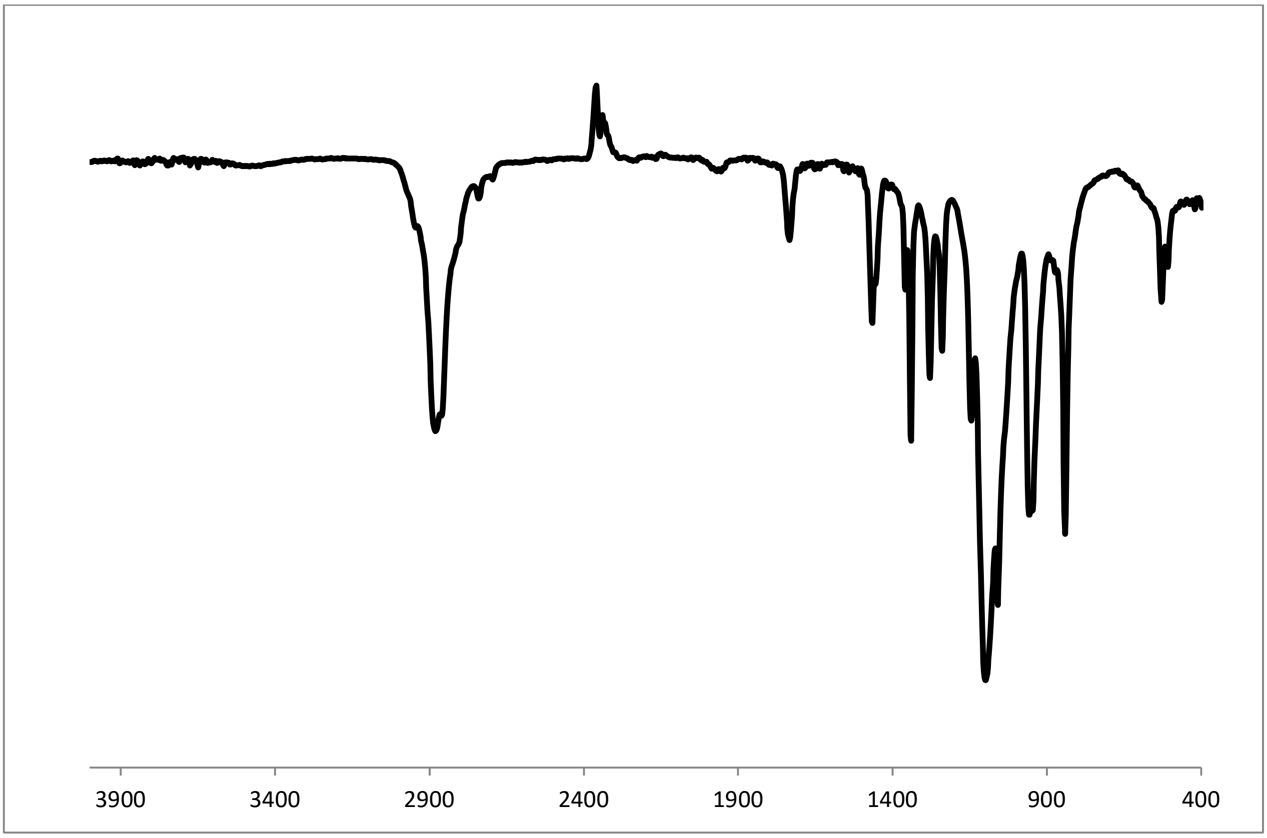

Figure 4b IR spectrum of P4 (neat)

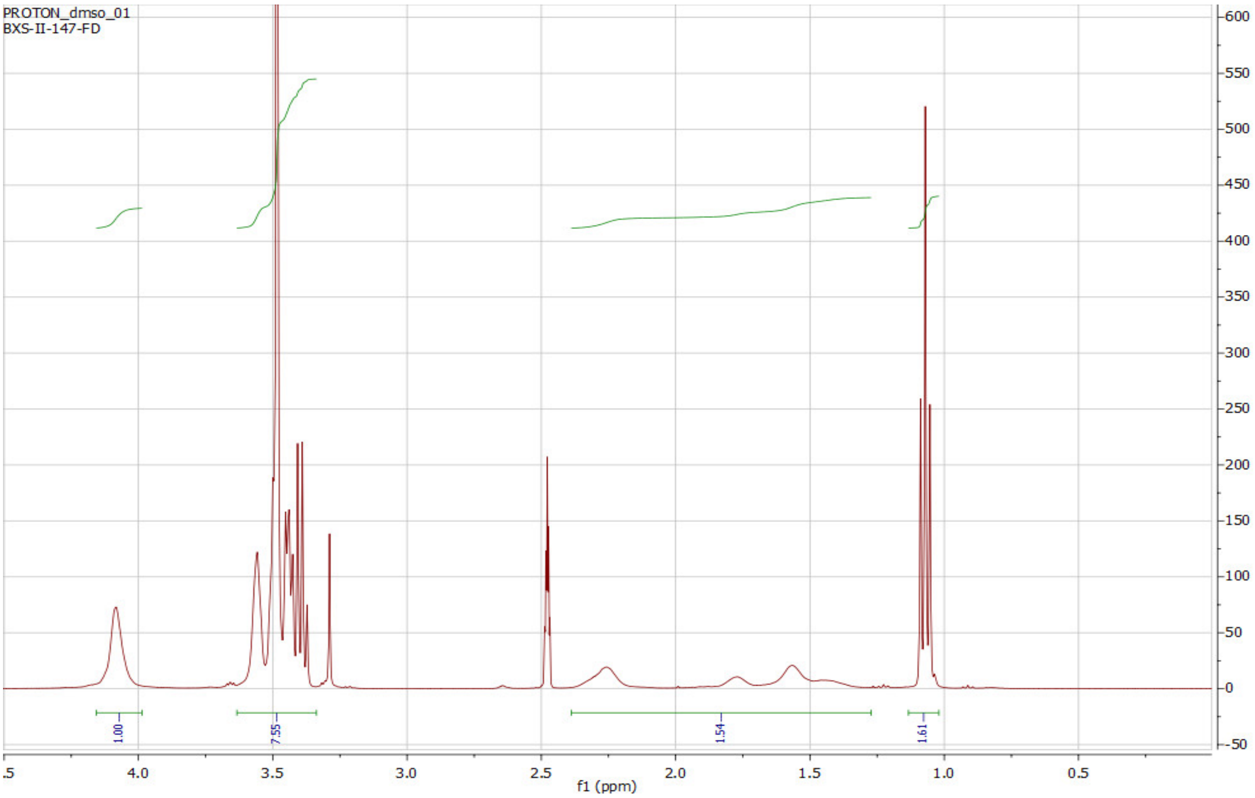

Figure 5a NMR spectrum of P5 (DMSO-D6)

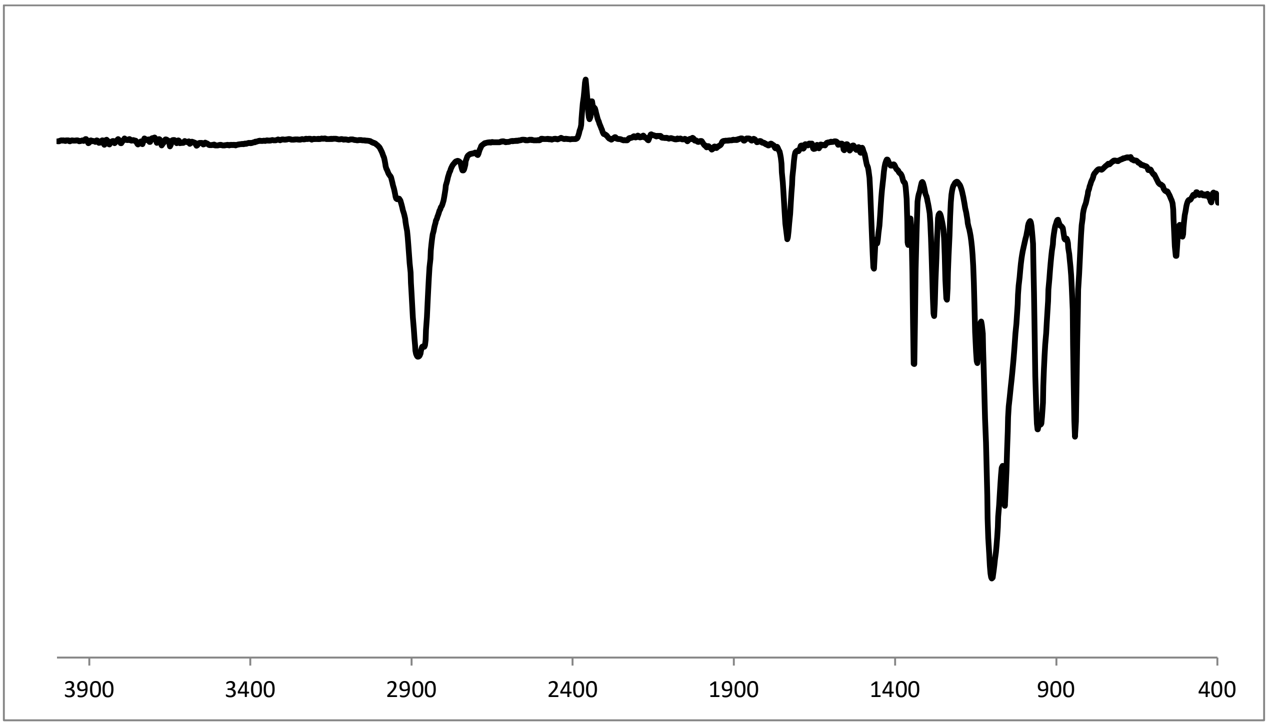

Figure 5b IR spectrum of P5 (neat)
